# Subspecialty Choices Among Medicine-Pediatrics Graduates: Results From a Four-Year National Program Director Survey

**DOI:** 10.7759/cureus.65665

**Published:** 2024-07-29

**Authors:** Anoop Agrawal, Daniel Wells, Michael Kisielewski, Savita Misra, Benjamin Doolittle

**Affiliations:** 1 Internal Medicine-Pediatrics, Baylor College of Medicine, Houston, USA; 2 Internal Medicine-Pediatrics, University of Tennessee Health Science Center College of Medicine, Memphis, USA; 3 Internal Medicine, Alliance for Academic Internal Medicine, Alexandria, USA; 4 Internal Medicine-Pediatrics, Yale School of Medicine, New Haven, USA

**Keywords:** med-peds graduates, subspecialty training, subspecialty, med-peds, career choices, medical resident education, med-peds residency

## Abstract

Background and objectives

Dual-trained medicine-pediatrics physicians (med-peds) play an important role in the healthcare ecosystem. Little is known about the subspecialty choices of med-peds residency graduates. This study aims to characterize the subspecialty choices of med-peds residency graduates.

Methods

The Medicine-Pediatrics Program Directors Association (MPPDA) administers an annual survey to the program directors of all med-peds residency programs accredited by the Accreditation Council for Graduate Medical Education (ACGME). This project represents aggregate survey data from 2020-2023.

Results

The number of program directors responding to the survey ranged from 80.8% (63/78) to 85.7% (66/77; mean response rate: 82.8%). About 465 of 1,245 (37%) graduates over the four years chose fellowship training, across 51 unique subspecialties. The top five selected pathways were: adult pulmonary and critical care 54 (11.6%), allergy and immunology 37 (7.9%), adult infectious diseases 30 (6.5%), adult cardiology 30 (6.5%), and pediatric cardiology 30 (6.5%).

Conclusions

Med-Peds residents pursue a diversity of subspecialty training and represent an important contribution to the subspecialty workforce. Improving combined subspecialty opportunities may increase participation by med-peds graduates and, in particular, may support the increasing need for pediatric subspecialists.

## Introduction

Dual-trained medicine-pediatrics (med-peds) physicians play an important role in the healthcare ecosystem [1]. More than 5,700 practicing physicians are dual-trained in the United States, representing one of the largest subspecialty committees in the American Academy of Pediatrics [2]. In a 2014 study of graduate career plans, 54% of med-peds-trained physicians pursued primary care, 22% hospital medicine, and 20% subspecialty medicine [3]. A follow-up study of unpublished data, aggregating data from 2017-2023 from the Medicine-Pediatrics Program Directors Association (MPPDA) annual survey of residency program directors, demonstrated that 16.9% pursue an internal medicine fellowship, 16.2% pursue private ambulatory or outpatient practice, 14.6% are med-peds hospitalists, and 10.2% pursue academic practice. Only 8.6% pursued pediatric fellowship, a notable contrast to their categorical pediatric peers. Based on data from the American Board of Pediatrics certification management system, 38% of graduates of pediatric residency programs pursue subspecialty fellowship [4]. The most common subspecialty fellowships in the 2022 match were neonatology (n=300), critical care (n=216), emergency medicine (n=216), hematology/oncology (n=163), and cardiology (n=162). Among internal medicine graduates, 64% pursue subspecialty fellowships, 22% pursue primary care, and 9% pursue hospitalist medicine [5]. Among those who choose subspecialty fellowship training, the most common are cardiology (n=1,199), pulmonary/critical care (n=781), and hematology/oncology (n=712) [6].

Little is known about the specific subspecialty choices among medicine-pediatric graduates, especially given the ever-evolving pressures in the healthcare system. Medicine subspecialists often earn a higher salary than pediatric subspecialists [7,8]. The debt burden, hospital-owned practices, and the growth of hospital medicine also influence the career choice of all residency graduates [9,10]. Within med-peds, graduates often have interdisciplinary interests in transitional care from childhood to adulthood, specific illnesses that cross the age spectrum such as diabetes mellitus and congenital heart disease, or encompassing issues such as global or public health [11]. These interests may drive subspecialty choices of med-peds graduates. This project represents the most comprehensive review of subspecialty choices among med-peds graduates, using four years of aggregate results from the MPPDA program directors’ annual survey.

## Materials and methods

Study setting and participants

The MPPDA is the organization of accredited combined med-peds programs in the United States and an affiliate organization of the Alliance for Academic Internal Medicine (AAIM), a professional association that represents over 12,000 internal medicine (IM) educators and administrators. The MPPDA research committee designs the survey with input from the MPPDA executive committee and its constituent committees. The voluntary survey is administered electronically to the program directors (PD) of all accredited med-peds residency programs in the United States through AAIM surveys and research personnel.

Instrument and data collection 

The annual survey collects data addressing multiple topics that generally remain static, including program structure and characteristics, clinical settings, curricular changes, and, as warranted, timely issues or themes. Each fall, the static question sections are re-reviewed for content validity and revised accordingly by the MPPDA research committee. That process includes identifying potentially problematic questions or items (e.g., responses to the previous year’s survey that obtained a high item non-response rate or a large number of “equivocal” responses such as “Do not know” or “Unsure”), revising them, and re-testing them with the committee and external pilot testers until all parties are in agreement with those modifications to the survey instrument. In December, AAIM surveys staff program the instrument in the Qualtrics surveys platform. The survey instrument thus varies from 70 to 80 questions based upon the addition of new issues or themes. The web survey is then pretested by the committee for functionality. Any revised questions or new thematic sections are also pilot-tested by a small number of MPPDA members with requisite subject matter experience, to improve upon content validity. 

Since 2020, the survey has included a section on med-peds resident graduates, including the number of residents who graduated during the previous academic year (AY) and the career fields selected by those residents. Program directors self-reported the subspecialty career for each graduate, using an open-text field. Further, respondents were asked to provide the subspecialty career choices of graduates who served an additional fifth year as chief residents (also via an open-text field). 

The 2020 through 2023 surveys were distributed to PDs (via an email invitation from the survey platform, including a unique survey participation URL for each PD) from all MPPDA member residency programs (n=77 to 79), effectively representing 100 percent of all programs with Accreditation Council for Graduate Medical Education (ACGME) “continued accreditation” at the time of each study. The survey landing page served as the study’s informed consent page. No incentives for participation were offered.

The 2020 through 2023 survey studies were deemed exempt by Pearl IRB (45CFR46.104(b)(2):(2) (see Appendix 1 for each study number) and were fielded from January through early March. At least three email reminder messages were sent to nonrespondents. Only AAIM survey staff (MK and/or SM) had access to the survey software during fielding. At survey closure, the study dataset was appended with limited, publicly available IM and med-peds residency program data from the ACGME and the American Board of Internal Medicine (ABIM). All responses were then de-identified, and the study dataset was restricted to project personnel via a secure networked file location using 256-bit encryption. 

Statistical analysis

Data analysis for the annual surveys was conducted in Stata (StataCorp. 2019. Stata Statistical Software: Release 16. College Station, TX: StataCorp LLC) by MK and/or SM. The residency program type was obtained from the American Medical Association fellowship and residency electronic interactive database access system online, through a data license [12]. Residency program director appointment year (and select characteristics) was obtained from the ACGME accreditation database system online (public) [13].

To describe the statistical representativeness of the survey responses, essential characteristics of responding programs (e.g., PD appointment year) were compared to the complete survey population using variables from the third-party data sources described above. Fisher’s exact test was used to test for associations between respondents and nonrespondents for any categorical variables; Welch’s t-test (with unequal variances) was used for comparisons based on continuous variables. Statistical significance was designated using an alpha level of p≤0.05. Multivariate tests (significance was designated at p≤0.01, due to the sensitivity of comparing multiple survey years in a dataset of finite size) were used to compare the mean percentage of reported med-peds graduates across each survey year. Responses to the open-ended text field for fellowship training were coded by AA into discretely identifiable fellowships (e.g., pediatric cardiology, combined infectious disease, adult endocrinology) and aggregated. For this study, the fields of allergy and immunology as well as palliative medicine were categorized as a combined med-peds fellowship, given that the training involves exposure to both adult and pediatric populations. There was no need for multiple coders or inter-rater reliability testing because the vast majority of open-text responses consisted of unequivocal reporting (e.g., adult pulmonology”).

## Results

The number of program directors completing the survey ranged from 80.8% (63/78) to 85.7% (66/77; mean response rate: 82.8%). There was no statistical under- or over-representation of respondents vs. non-respondents based on residency program and PD characteristics (e.g., U.S. census region, PD appointment year) that most closely defined the survey population (Table 1).

**Table 1:** Statistical representativeness of the survey responses: 2020-2023 MPPDA annual surveys (aggregated) MPPDA: Medicine-Pediatrics Program Directors Association; AMA-FREIDA: American Medical Association Residency and Fellowship Database; ACGME: Accreditation Council for Graduate Medical Education; ABIM: American Board of Internal Medicine; VA: Veterans Affairs; SD: standard deviation. ^a^Fisher’s Exact Test (two-sided) used to compare categorical variables ^b^From the Alliance for Academic Internal Medicine - MPPDA membership database. ^c^Excludes programs from U.S. territories, due to small cell sizes/data confidentiality. Thus, totals will be slightly less than those reported for each column. ^d^Welch’s T-Test with unequal variances. ^e^Not collected for the 2020 and 2021 Annual Surveys. Alpha level: p≤0.05

	Respondents (n=259)	Nonrespondents (n=54)	Total (n=313)	
Characteristic	No. (Column %)	No. (Column %)	No. (Column %)	p-value^a^
Program director self-reported gender^b^				
Female	126 (48.6)	28 (51.9)	154 (49.2)	0.702
Male	133 (51.4)	26 (48.1)	159 (51.0)	
Residency program type (AMA-FREIDA)				
University-based	224 (86.5)	41 (79.3)	265 (84.7)	0.528
Community-based, university-affiliated	35 (13.5)	13 (24.1)	48 (15.3)	
Program U.S. Census Bureau Region^c^				
Northeast	65 (25.5)	5 (9.3)	70 (22.7)	0.407
Midwest	79 (31.0)	13 (24.1)	92 (29.8)	0.544
South	87 (34.1)	32 (59.3)	119 (38.5)	0.174
West	24 (9.4)	4 (7.4)	28 (9.1)	0.821
Characteristic	Mean (SD)	Mean (SD)	Mean (SD)	p-value^d^
Program director appointment year (ACGME)^e^	2013.6 (6.7)	2013.3 (9.8)	2013.6 (7.2)	0.702

**Figure 1:**
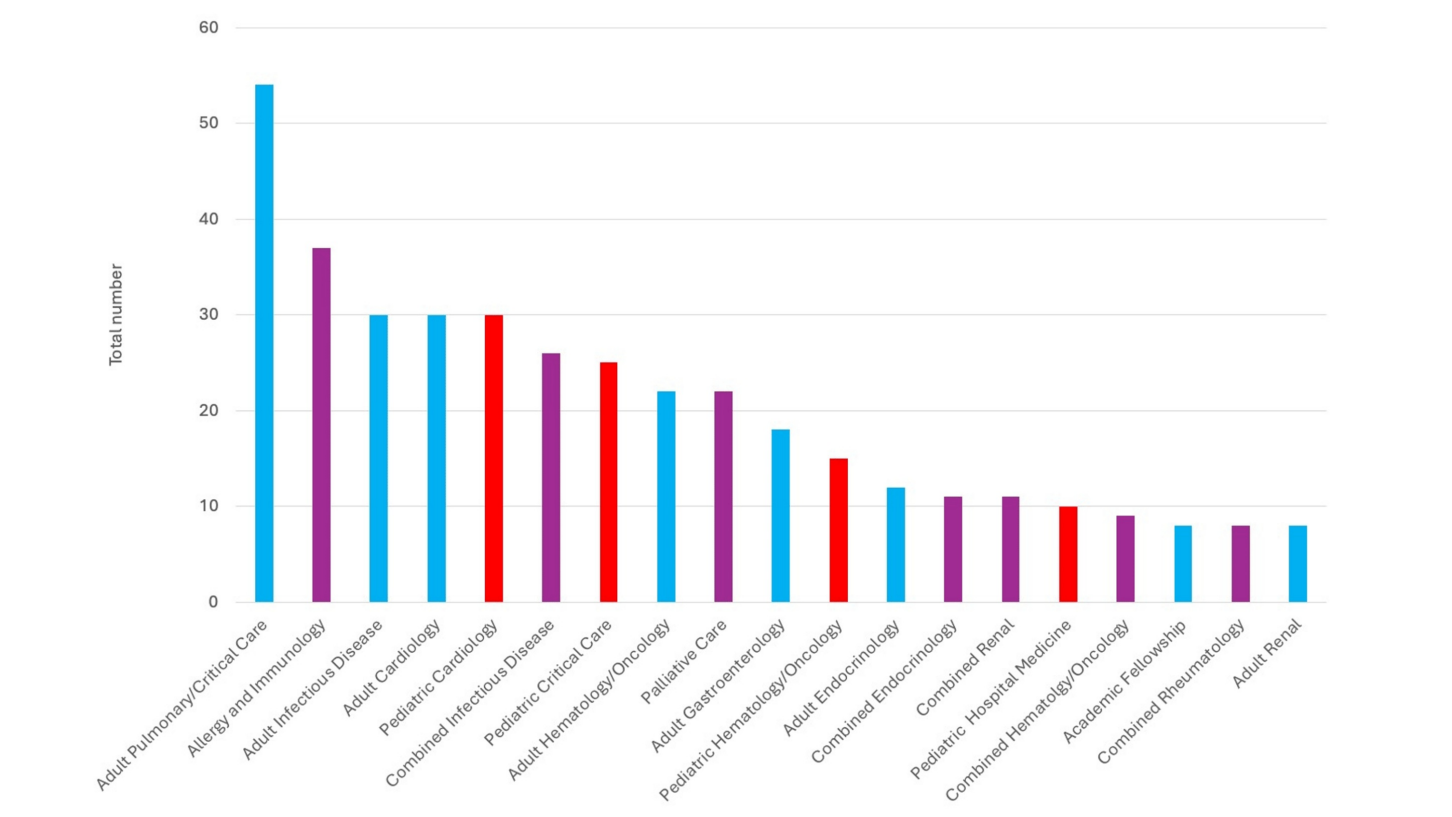
Subspecialty choices amongst med-peds graduates from 2020 to 2023 blue bar: internal medicine fellowship; red bar: pediatric fellowship; purple bar: combined fellowship

Over the four survey years, there was little variability in the mean percentage of med-peds graduates (n=1,294) by PD-reported career field (p>0.01 for all multi-year comparisons). Including graduates pursuing career fields following a fifth-year chief residency, 467 out of 1,294 (36%) graduates were reported to have chosen subspecialty training. Within those choosing a subspecialty, internal medicine fellowships were selected by 48% of graduates as compared to 26% each for pediatric or combined med-peds fellowships. The percentage of graduates choosing subspecialty careers had consistently held at 34 to 35% per year, however, 2023 saw an increase to 40% (Table 2). The top-five reported pathways were: adult pulmonary and critical care 54 (11.6%), allergy and immunology 37 (7.9%), adult infectious diseases 30 (6.5%), adult cardiology 30 (6.5%), and pediatric cardiology 30 (6.5%) (Figure 1). Among graduates who chose career paths outside of ambulatory practice and hospital medicine, 51 uniquely identifiable subspecialties and alternative career paths were reported (Table 3).

**Table 2:** Graduates’ career choices as a percent of all career choices reported: 2020-2023 MPPDA annual surveys MPPDA: Medicine-Pediatrics Program Directors Association; IM: Internal Medicine; Peds: Pediatrics. “No.” represents the number of graduates reported to select each career field; “Total graduates” represents the total number of graduates reported and therefore is the denominator for each column percentage. ^a^Multivariate test of means across all survey years: Hotelling T2 with three degrees of freedom. ^b^From 2021 onward, “Private Practice” was presented as “Private Ambulatory / Outpatient practice.” ^c^In 2021, the response option “Global Health” was added. ^d^Open-text responses to “Other” were coded to inform the career field choices reported in this manuscript. Alpha level: p≤0.01

Career Choice	Survey Year (n = Number of Respondents to Question)					
	2020 (n=65)	2021 (n=65)	2022 (n=63)	2023 (n=66)		
	No. (%)	No. (%)	No. (%)	No. (%)	Hotelling T^2a^	p-value
Private Practice^b^	56 (17.3)	48 (14.7)	51 (16.4)	40 (12.0)	1.64	0.651
Academic Practice	26 (8.0)	41 (12.6)	30 (9.6)	30 (9.0)	1.37	0.714
Med-Peds Fellowship	27 (8.3)	27 (8.3)	34 (10.9)	33 (9.9)	0.91	0.823
IM Fellowship	51 (15.7)	53 (16.3)	51 (16.4)	71 (21.3)	2.16	0.542
Peds Fellowship	33 (10.2)	32 (9.8)	25 (8.0)	30 (9.0)	0.77	0.857
Chief Resident	29 (9.0)	30 (9.2)	31 (10.0)	29 (8.7)	0.05	0.997
Med-Peds Hospitalist	43 (13.3)	53 (16.3)	41 (13.2)	50 (15.0)	0.87	0.834
IM Hospitalist only	34 (10.5)	27 (8.3)	34 (10.9)	34 (10.2)	0.77	0.858
Peds Hospitalist only	3 (0.9)	3 (0.9)	1 (0.3)	2 (0.6)	1.62	0.657
Global Health^c^	--	4 (1.2)	3 (1.0)	5 (1.5)	0.42	0.811
Other^d^	22 (6.8)	8 (2.5)	10 (3.2)	9 (2.7)	2.41	0.494
Total graduates	324	326	311	333		

**Table 3:** All subspecialty and alternative training choices *Training pathways designated as a combined fellowship

Subspecialty and alternative training choices	
Addiction Medicine	Global Health
Adolescent	Health Services Research
Adult Academic Fellowship	Infectious Disease/Critical Care
Adult Cardiology	Informatics
Adult Cardiology/Critical Care	Med-Peds Hospital Medicine*
Adult Endocrinology	Neonatology
Adult Gastroenterology	Obstetrics Medicine
Adult Hematology/Oncology	Obesity Medicine
Adult HIV	Palliative Care*
Adult Infectious Disease	Pediatric Academic Fellowship
Adult Pulmonology	Pediatric Cardiology
Adult Pulmonology/Critical Care	Pediatric Critical Care
Adult Renal	Pediatric Dermatology
Adult Rheumatology	Pediatric Emergency Medicine
Allergy and Immunology*	Pediatric Gastroenterology
Combined Critical Care*	Pediatric Hematology/Oncology
Combined Endocrinology*	Pediatric Hospital Medicine
Combined Gastroenterology*	Pediatric Infectious Disease
Combined Hematology/Oncology*	Pediatric Pulmonology
Combined Infectious Disease*	Pediatric Renal
Combined Pulmonology*	Pediatric Rheumatology
Combined Renal*	Sickle Cell
Combined Rheumatology*	Sleep medicine
Developmental Medicine	Sports Medicine
Epidemic Intelligence Service	Transition Medicine*
Geriatrics	

## Discussion

During our measured timeframe, med-peds graduates entered 51 unique and alternate career paths, representing a large and increasing part of the subspecialty workforce (Table 3). Compared to results from the 2014 workforce survey in which 20% of med-peds graduates were subspecialty trained, more than a third of graduates are now opting into fellowship training [3]. Both pediatric and adult critical care have been amongst the top destinations for med-peds graduates during that time, more so in internal medicine (Figure 1). Our survey results demonstrate that the fellowship choices of med-peds graduates mostly mirror the popular fellowship choices of their categorical peers [14]. Anecdotally, several residents have been able to work with individual fellowship programs to create and enter combined specialties, most prominently in the non-procedural specialties of endocrine, infectious diseases, and nephrology. However, the numbers are lower than we would postulate based on career-planning conversations with our trainees, and the number of combined specialties has remained static. Inherently combined specialties such as allergy and immunology and palliative care have remained popular choices among graduates. Sparsely represented in the career destinations of med-peds graduates are combined fellowships in the procedural specialties of pulmonary/critical care and gastroenterology.

Although med-peds graduates are fully certified in both internal medicine and pediatrics and spend equal time in each during training, med-peds residents have matched into adult specialties more often than pediatric specialties by nearly a 2:1 ratio. Our data do not allow us to explain this discrepancy, although this tracks with the concerning national trend of decreasing pediatric fellowship fill rates [3,15]. As a combined residency, med-peds residents are completing four years of training rather than three, perhaps making longer fellowships less desirable. Currently, many pediatric fellowships have ACGME research requirements that often lead to an extra year of training compared to their IM counterparts. Complicating the extra year of training, the Doximity/Curative 2023 physician compensation report [7] found that many pediatric subspecialists in three-year fellowship programs (e.g. endocrine, infectious disease, rheumatology, hematology/oncology, nephrology) have lower reimbursement rates than general pediatricians. We postulate that the lower reimbursement rates as well as a standard three-year fellowship involving a research year might be less appealing for graduates of a combined, and, thus, longer residency training program. 

Despite the transition of care for patients with chronic pediatric illnesses remaining a weak point in the healthcare system, the number of combined fellowships has remained static during this timeframe and remains non-standardized [16-18]. The high front-end work by residents and programs for a combined fellowship remains challenging and is a likely reason for the low rates of med-peds residents pursuing this route [19]. We postulate that the motivation to care for patients across the age spectrum is desired, as evidenced by 29% of graduates entering self-created or inherently combined specialties. Standardizing fellowship options at both the ACGME and institutional levels and decreasing the workload on residents to develop combined fellowship opportunities would likely be desirable among med-peds graduates. It is the experience of the authors of this paper that a standardized route to a combined fellowship would be appealing to many residents, although there currently are no data that measure med-peds resident desire for combined fellowship, and this could be an area of future study.

To our knowledge, this study is the first to cite specific destinations for med-peds residents who pursue fellowship training. We believe that these survey findings demonstrate the versatility of med-peds graduates and that all subspecialties are benefitting from the unique training that med-peds provide. Although many med-peds residents desire to care for both children and adults after graduation, there is still no standardized pathway for residents to enter combined fellowships beyond those that are inherently combined, such as allergy and immunology. This is an opportunity for national growth to fill the gaps in the transitional time.

Limitations

Our study has several limitations. The first is the survey design itself, which did not collect specific data about residents beyond their subspecialty choices. Thus, it is not possible to determine whether resident demographics such as gender, age, or ethnicity, or certain program characteristics such as geographic region, might be associated with fellowship choices. The second limitation is that our study is based on a standardized survey of med-peds program directors and relies on self-reporting, which might have introduced some degree of recall bias or classification error. However, the likelihood of either error is low, given the timing of the survey in relation to graduation, the small size of most med-peds programs, and the relative continuity in program directors of record. A third limitation is that some fellowships are difficult to classify because they are non-standard (e.g., obesity medicine, hematology), mostly non-clinical (e.g., informatics), or not specific to a single, readily describable population (e.g., sickle cell, obstetrics). Additionally, the survey only captures the initial career choice upon residency graduation. Currently, there is no mechanism for capturing graduates who delay entering fellowship for any variety of reasons. Anecdotally, we believe this is a very small number of individuals. Lastly, we have no data from this survey to indicate the desired destinations of graduates who either matched into a specialty that was not their primary choice or did not match. Nevertheless, the steadily high and representative response rate of the survey during the timeframe studied suggests that the results are generally consistent.

## Conclusions

This study represents the largest aggregate report of med-peds subspecialty destinations. The unique niche of dual training allows for flexibility and plays an important role in filling healthcare gaps. Dual-trained specialists allow for cross-fertilization between departments, innovation, and care of vulnerable populations who are often overlooked. The number of med-peds residents entering fellowships has grown and constitutes nearly one-third of the graduates over the most recent three-year period. Despite this unique niche and the flexibility that med-peds training affords, med-peds residents tend to choose internal medicine specialties more than pediatric specialties. Additionally, the lack of standardization of combined specialties likely results in fewer med-peds residents who pursue combined fellowship training. We believe that standardization of combined fellowship training and dedication to funding would yield a higher number of combined specialists, which would in turn help fill transitional voids and increase the number of specialists in pediatric subspecialties.

## Appendices

Appendix 1 

**Table 4:** MPPDA Internal Medicine – Pediatrics Residency Program Directors annual survey response rates and human subjects research protections study numbers and determinations: 2020 to 2023

Survey Year	Total respondents / Total possible respondents (%)	Study Number (#) and Determination
2020	65/79 (82.3)	#20-AAIM-110; exempt, Pearl IRB (U.S. DHHS OHRP #IRB00007772) under FDA 21 CFR 56.104 and 45CFR46.104(b)(2): (2)
2021	65/79 (82.3)	#20-AAIM-114; exempt, Pearl IRB (U.S. DHHS OHRP #IRB00007772) under FDA 21 CFR 56.104 and 45CFR46.104(b)(2): (2)
2022	63/78 (80.8)	#21-AAIM-121; exempt, Pearl IRB (U.S. DHHS OHRP #IRB00007772) under FDA 21 CFR 56.104 and 45CFR46.104(b)(2): (2)
2023	66/77 (85.7)	#22-AAIM-119; exempt, Pearl IRB (U.S. DHHS OHRP #IRB00007772) under FDA 21 CFR 56.104 and 45CFR46.104(d)(2)
Note: MPPDA: Medicine-Pediatrics Program Directors Association.		
